# Potential Roles of Metals in the Pathogenesis of Pulmonary and Systemic Hypertension

**DOI:** 10.7150/ijbs.85590

**Published:** 2023-09-25

**Authors:** C. Danielle Hopkins, Caitlin Wessel, Oscar Chen, Karim El-Kersh, Matthew C. Cave, Lu Cai, Jiapeng Huang

**Affiliations:** 1 Department of Anesthesiology and Perioperative Medicine, University of Louisville School of Medicine, Louisville, KY, USA.; 2 Department of Internal Medicine, Division of Pulmonary Critical Care and Sleep Medicine, University of Nebraska Medical Center, Omaha, NE, USA.; 3 Division of Gastroenterology, Hepatology, and Nutrition, Department of Medicine, University of Louisville School of Medicine, Louisville, KY, USA.; 4 The Center for Integrative Environmental Health Sciences, University of Louisville, Louisville, KY, 40202, USA.; 5 Department of Pharmacology and Toxicology, University of Louisville School of Medicine, Louisville, KY, USA.; 6 Department of Biochemistry and Molecular Genetics, University of Louisville School of Medicine, Louisville, KY, USA.; 7 The Transplant Program at UofL Health - Jewish Hospital Trager Transplant Center, Louisville, KY, USA.; 8 Pediatric Research Institute, Department of Pediatrics, University of Louisville School of Medicine, Louisville, KY, USA.; 9 Department of Radiation Oncology, University of Louisville School of Medicine, Louisville, KY, USA.; 10 Cardiovascular Innovation Institute, Department of Cardiovascular and Thoracic Surgery, University of Louisville School of Medicine, Louisville, KY, USA.

**Keywords:** Pulmonary artery hypertension, Pulmonary hypoxia, Right ventricle dysfunction, Trace elements, Mineral homeostasis, Heavy metals, Non-essential metals, Mitochondrial dysfunction, Hypertension

## Abstract

Pulmonary and systemic hypertension (PH, SH) are characterized by vasoconstriction and vascular remodeling resulting in increased vascular resistance and pulmonary/aortic artery pressures. The chronic stress leads to inflammation, oxidative stress, and infiltration by immune cells. Roles of metals in these diseases, particularly PH are largely unknown. This review first discusses the pathophysiology of PH including vascular oxidative stress, inflammation, and remodeling in PH; mitochondrial dysfunction and metabolic changes in PH; ion channel and its alterations in the pathogenesis of PH as well as PH-associated right ventricular (RV) remodeling and dysfunctions. This review then summarizes metal general features and essentiality for the cardiovascular system and effects of metals on systemic blood pressure. Lastly, this review explores non-essential and essential metals and potential roles of their dyshomeostasis in PH and RV dysfunction. Although it remains early to conclude the role of metals in the pathogenesis of PH, emerging direct and indirect evidence implicates the possible contributions of metal-mediated toxicities in the development of PH. Future research should focus on comprehensive clinical metallomics study in PH patients; mechanistic evaluations to elucidate roles of various metals in PH animal models; and novel therapy clinical trials targeting metals. These important discoveries will significantly advance our understandings of this rare yet fatal disease, PH.

## 1. Introduction

Pulmonary hypertension (PH) is currently defined as an increased mean pulmonary arterial pressure greater than 20 mmHg measured by right heart catheterization at rest. It can be further divided into pre-capillary and post-capillary PH based on other hemodynamic parameters, namely pulmonary arterial wedge pressure (PAWP) and pulmonary vascular resistance (PVR). Pre-capillary PH, or pulmonary arterial hypertension (PAH), is defined as PAWP ≤ 15 and PVR > 2 Woods Units (WU). Conversely, isolated post-capillary PH uses PAWP > 15 and PVR ≤ 2 WU as its defining parameters and is associated with left-sided heart disease. Combined pre- and post-capillary PH is a combination of the two previous definitions with PAWP > 15 and PVR > 2WU 1, 2. While the precise mechanism for the development of PH remains unknown, endothelial mitochondria have been shown to play crucial roles in the pulmonary circulation under both healthy and pathologic conditions. One of their many cellular functions is forming reactive oxygen and nitrogen species (ROS, RNS) 3, which have been shown to affect the cellular pathways of pathogenic oxidative stress in PH.

Currently there is no consensus regarding how or why endothelial mitochondrial dysfunction occurs. One proposed mechanism comes from the interaction between the mitochondria and metals. Non-essential metal pollution from industrialization and urbanization, impacts everyone through the contamination of water, soil, and the atmosphere 4-6. Introduction of non-essential metals into the environment occurs in several ways, including but not limited to mining, smelting, landfills, livestock, and air pollution. In the setting of PH, non-essential metals have been shown to disrupt pulmonary artery endothelial and smooth muscle cell (PAEC and PAMSC) mitochondrial functions and increase ROS production 3. In addition, inhalation or ingestion of these metals can cause nucleic acid, protein, and/or lipid dysfunction 6. Evidence further supports metal exposure as a risk factor for the development of cardiovascular disease (CVD) specifically lead (Pb), arsenic (As), copper (Cu), and cadmium (Cd). Consequently, these metal exposures may also be risk factors for the development of PH 1, 2, 7.

Although PH and systemic hypertension (SH) shared some similarity, they are two significantly different diseases in terms of etiology, risk factors, pathogenic target organs and pathogenic mechanisms (Table 1). Considering that the pathogenic effects of several metals on SH have been well investigated, a brief update on this research will be provided before introduction of the potential involvement and impact of metals on PH. Therefore, the aim of this review is to assess the evidence surrounding nonessential and essential metals, mitochondrial dysfunction, and the subsequent ROS/RNS production in the pathogenesis of SH and PH.

## 2. Brief introduction of the pathophysiology of PH

### 2.1 Vascular oxidative stress, inflammation, and remodeling

**Figure 1:**
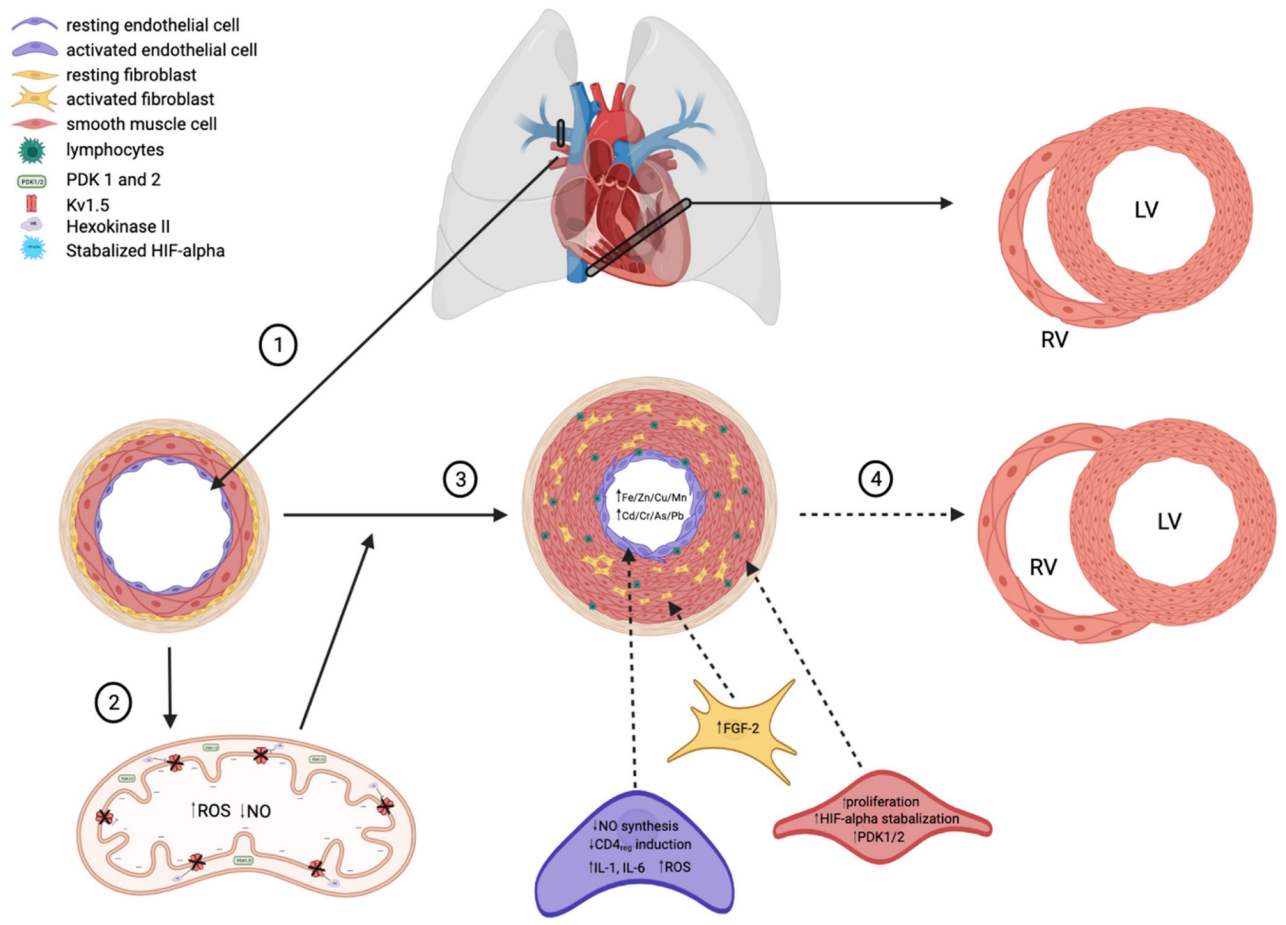
The are multiple hypothesized factors that contribute to the decreased lumen diameter characteristic of PAH, many centered around the development of a hypoxic state and increased concentration of heavy metals. **(1)** Healthy pulmonary arteries are highly compliant to meet the homeostatic demands of the heart (RV=right ventricle, LV=left ventricle) and peripheral circulation. **(2)** In states of low oxygen availability, HIF-alpha is stabilized and thus activated. HIF-alpha stabilization upregulates PDK1/2 expression in apoptotic-resistant pulmonary endothelial and smooth muscle cells, thus decreasing oxidative phosphorylation and promoting the production of ROS. A shift towards increased ROS production in mitochondria consequently decreases NO synthesis, thus allowing for unregulated vasoconstriction of pulmonary arteries. **(3)** Idiopathic PAH presents with increased lymphocyte infiltration of endothelial cells, resulting in activation and an increased in inflammatory cytokine release (i.e., IL-1, IL-6). IL-1 upregulates FGF-2, which promotes smooth muscle cell proliferation and thus decreased lumen size. **(4)** Decreased pulmonary artery compliance over time leads to cardiac right ventricular remodeling, a complication frequently seen in PAH patients.

The pathophysiology of PH is the combination of endothelial cell damage, immune dysregulation, oxidative stress, and maladaptive metabolism, eventually leading to remodeling of the cardiopulmonary vasculature, as shown in **Figure 1**. The chronic and mechanical stress experienced by endothelial cells leads to inflammation, and infiltration by immune cells that potentiates the inflammatory process. Cellular and molecular responses from many signaling pathways cause pulmonary vasoconstriction, remodeling, and in situ thrombosis, which lead to increased vascular resistance and pulmonary artery pressure. The right ventricular (RV) afterload increases in PH eventually leads to RV remodeling, right heart failure, and death 2, 8, 9.

Increased blood flow through the pulmonary vasculature impacts pulmonary arterial fibroblasts (PAFBs), PAECs, and PASMCs. Each of these cell types communicate via paracrine and mechanical signaling. Fluid shear stress not only impacts these cells individually but affects them as a network 10-12. These results in overproduction of extracellular membrane proteins leading to elastin and collagen deposition, increased stiffness of the pulmonary vasculature, activated fibroblasts, and increased extracellular matrix (ECM) gene expression and protein deposition. The subsequent proliferation of these cells and ECM accumulation result in the remodeling and angiogenesis 10, 12. Inflammatory cytokines released from both PAECs and inflammatory cells induce interleukin 1 or 6 (IL-1 or IL-6) and fibroblast growth factor-2 (FGF-2) expression to regulate the proliferation of PASMCs and PAFBs 9. The resulting fibrosis and smooth muscle hyperplasia obstruct the vascular lumens and lead to rise in vascular wall pressure, pulmonary vascular resistance (PVR), and pulmonary artery pressure 8, 9, 12.

Bone morphogenetic proteins (BMPs), as a member of the TGF-β superfamily of ligands, are involved in paracrine signaling. BMP type II (BMPR2) is a serine/threonine receptor kinase that binds to BMPs. BMPR2 is the first gene identified in families with PAH and its mutation accounts for around 70% to 80% of heritable PH cases 13, 14. The binding of BMPR2 to BMP, which plays important roles in a wide range of signal pathways involved in cellular differentiation, growth, and apoptosis. In the canonical (or Smad-mediated) signaling pathway, BMPR2 recruits, complexes and phosphorylates BMP type 1 receptors that in turn phosphorylate SMADs (R-SMADs) pathways. In addition, BMPR2 also activates a few non-canonical pathways, including p38 mitogen-activated protein kinase, extracellular signal-regulated kinase, and phosphoinositide 3-kinase /Akt signaling pathways 15. Therefore, dysregulation and loss of function of BMPR2 are essential in PH vascular inflammation and remodeling via various mechanisms. For instance, mutated BMPR2 in PAECs of pulmonary vascular intimal layer increases the apoptotic rate, causes release of pro-inflammatory mediators like TNF-α, enhances production of TGF-β and FGF, and induce endothelial dysfunction 13-15.

Endothelial dysfunction decreases endothelial nitric oxide (NO) synthase (eNOS) phosphorylation, decreases NO generation, increases vasoconstriction, and causes higher pressure in the pulmonary vasculature. BMPR2 dysregulation causes overproliferation and overmigration of PASMCs in the tunica media and disrupts the expression of vascular endothelial growth factor (VEGF) that reduces vascular formation and function in vascular adventitia. All these changes cause pulmonary vasculature to be more susceptible to hypoxia 14. Endothelial dysfunction and vascular inflammation can result from increased oxidative stress and irreversible oxidative damages. Oxidative stress markers such as malondialdehyde, total antioxidant capacity, superoxide dismutase (SOD), and catalase activities were increased and associated with adverse clinical outcomes in PH 16, 17. NO is responsible for the vasodilation of vasculature in the pulmonary circulation. Decreased activities of eNOS result in lower levels of local NO and decreased bioavailability of NO leads to local vasoconstriction along with inflammation, and thrombosis 18-20. Uncoupled eNOS does not generate NO, instead generates superoxide during the progression of PH. Increased superoxide can interact with NO to further reduce NO availability and form more reactive species peroxynitrite (ONOO) 18-20. Patients with idiopathic PAH (IPAH) demonstrate low NO levels when compared to healthy controls 21. The role of oxidative stress in PH was supported by that in vivo administration of memetic of SOD and CAT reversed pulmonary vascular remodeling in rats 22.

### 2.2 Mitochondrial dysfunction and metabolic changes

Oxidative stress and ROS result in mitochondrial dysfunction 23-25. The connection between metabolic changes and PH is referred to as the “metabolic theory” of PH or the Warburg Effect 26-28. Warburg Effect, first described in tumor cells, is well illustrated in the context of PH. Due to the accumulation of ROS and stabilization of hypoxia-inducible factor (HIF)-1α, mitochondria shift the bulk of ATP production from the more efficient oxidative phosphorylation to glycolysis—regardless of the presence of oxygen 29. In this case, even in the absence of true hypoxia, transcription factors are activated, and contribute to the metabolic reprogramming 29.

This metabolic dysregulation and mitochondrial dysfunction inhibit mitochondria-mediated apoptosis despite the release of pro-apoptotic factors (i.e.: cytochrome c), due in part to hyperpolarization of the mitochondrial membrane 3, 29, 30. In the setting of PH, HIF-1α mediates the metabolic shift through upregulation of pyruvate dehydrogenase kinases 1 and 2 that inhibit the conversion of pyruvate to acetyl-CoA via phosphorylating and consequently inactivating pyruvate dehydrogenase (PDH). PDH regulates entry of acetyl-CoA into the Krebs Cycle, so PDH inactivation shifts cells into anaerobic respiration and establishes a pseudo-hypoxic state 3, 23, 25, 30.

### 2.3 Ion channel and its alterations in the pathogenesis of PH

The conversion of superoxide anions to hydrogen peroxide by MnSOD affects potassium (Kv) channels—such as those encoded by KCNK3— in the plasma membrane of healthy cells. KCNK3 encodes a K leak channel that regulates smooth muscle contraction via establishment of a concentration gradient. Kv channels not only control the major influx and efflux of potassium ions, but also the calcium (Ca) ions of the PASMCs, which are required for normal function. Both experimental models and human PH demonstrate downregulation of Kv channels. Kv1.5 channels are especially susceptible, as they are expressed in the small blood vessels typically affected by PAH vascular remodeling 30, 31. The downregulation and internalization of these Kv channels leads to a buildup of intracellular potassium efflux that is an inhibitor of caspases as an initiator for apoptosis. Apoptotic-resistant cells play a key role in the vascular lumen obliteration, so downregulating these channels may contribute to that portion of the pathophysiology 3, 30. In a rat model, blockage of KCNK3 increased smooth muscle proliferation, inflammation, and arterial remuscularization 32, 33. Consistent with these results, KCNK3 gene mutations and its incomplete function were found in PH patients 13, 21.

Piezo1, a cation channel that responds to mechanical stress, is upregulated in PAECs of IPAH patients and animal models of PH. This channel mediates Ca^2+^ and sodium influx in response to stretch in the vascular wall. In animal models, Piezo1 proteins and mRNA were upregulated in isolated pulmonary artery when compared to controls. Blocking Piezo1 signaling with an intraperitoneal injection of GsMTx4, a Piezo1 channel blocker, improved the PH in mouse models 34, 35. These cells undergo transformation to mesenchymal cells through loss of endothelial cell markers 10, 12, 34. PASMCs can detect shear stress through signaling of endothelial cells or directly in the case of endothelial cell damage. In PH patients, PASMCs exhibit hypersensitivity to fluid shear stress. This results in increased Ca^2+^ influx into the cytosol which increases the expression of mechanosensitive channels in the transient receptor potential (TRP) channel superfamily and leads to vasoconstriction. As one of the TRP channel superfamily members, TRPM4 is a Ca-activated nonselective cation channel and TRPM7 is magnesium -permeable and Mg-modulated cation channels. TRPM7 and TRPV4 channels had increased expression in IPAH patient when compared to controls. Inhibiting these channels decreases the vasoconstriction and vascular remodeling in PH patients 10, 12, 34. Several elegant reviews of gene expression and function as well as details for each of these channel proteins have been published 36, 37.

### 2.4. PH-associated RV remodeling

The above discussions are the main pathogenic changes and associated mechanisms in the PAEC death and differentiation, PAMSC proliferation, and fibrotic responses. These changes lead to the remodeling of the arterial wall, arterial stiffness and loss of the normal vascular dilation and contraction (Figure 1). In fact, these changes result in the high resistance for the RV to pump blood into the lungs via the pulmonary artery. Therefore, the pathophysiology of PH often results in RV remodeling and right heart failure due to the continuously elevated pressures. The RV myocyte remodeling proceeds in a stepwise fashion. During hypertrophic growth, mitochondrial dysfunction and HIF-1α expression are both observed 23, 38. RV remodeling necessitates multiple metabolic alterations. In patients who are genetically predisposed to PH, such as BMPR2 haploinsufficient patients, their predisposition stems from chronic inflammation and is linked to inherited mitochondrial dysfunction 39. Rats with mutant BMPR2 gene showed cellular and molecular dysfunctions in both lung and heart, as seen in human PAH 40.

Cardiac hypertrophy characteristic of severe PAH is also affected by oxidative damage. The GATA binding protein 4 (GATA4) transcription factor has been implicated in PASMC growth and proliferation. RV hypertrophy in response to hypoxia and PH increases GATA4 expression. In PASMCs, serotonin (5-HT) activates GATA4, and produces hydrogen peroxide, a potential source of ROS/RNS. Therefore, PH activates GATA4 pathway-mediated oxidative stress and RV cardiac remodeling and dysfunction 41-43.

In summary, oxidative stress result in mitochondrial dysfunction and leads to metabolic dysregulation. High energy requirements, potentially exacerbated by PH, increase production of various free radicals that damage mitochondrial membranes and proteins involved in metabolic functions. Therefore, the pathogenesis of PH is significantly different from SH (Table 1).

## 3. Metal metabolism, essentiality and its effects on systemic hypertension and pulmonary vascular remodeling

### 3.1 Metal general features and essentiality for the cardiovascular system

Metals are categorized into different groups based on their various features: transient (transition) metals vs non-transition metals based on their chemical features; heavy metals vs non-heavy metals from atomic weight; and essential vs non-essential metals from their biological effects. Electrons of transition metals can participate in the formation of chemical bonds—in two shells instead of only one. Transition metals are shown in red font in **Table 2**. Heavy metals are those with either a high atomic weight or density 6. Essential metals are those required for humans, like iron (Fe) and Cu, and the latter is not at all necessary for life, like Cd, Pb, and mercury (Hg).

**Table 2** lists some commonly investigated non-essential and essential metals in the toxicological studies, with overlap among three categories. For instance, the heavy metals and transition metals include both non-essential and essential metals 6, 44. For humans, 10 essential metal ions are indispensable for life. These are divided into two groups: 4 'bulk elements' and 6 'trace elements.' The bulk elements are Na, K, Ca and Mg and called as “bulk” since these together constitute approximately 99% of the metal ion content in the human body. The 'trace elements' include manganese (Mn), Fe, cobalt (Co), Cu, Zn and molybdenum (Mo), and consist about 1% of body metal contents 44, 45.

Metal toxicity can be caused by both non-essential metals and essential metals. When introduced into the human, nonessential metals cause toxic effects. Essential metals can also be toxic when these metals accumulate to excessive levels systemically or in specific tissues or organs. It should be noticed that if the essential metal levels are too low, which is called metal deficiency, it can cause specific symptoms related to the specific essential metal 44. The toxicity caused by nonessential metals can occur directly or indirectly by displacement of essential metals in normal biological processes that may reduce antioxidant expression and activity with dysregulating redox reactions 159.

As mentioned above, mitochondria play an important role in ROS/RNS, ATP production, conversion, and transduction of information, and cell death process. The mitochondrial responses in exposure to heavy metals has been discussed for a few decades. To date, several metals are known to distribute to, and accumulate in, the mitochondria where trace amounts of metals may be required for normal functioning of biological membranes as well as enzyme systems, whereas excess levels may be toxic 46-48.

**Figure 2:**
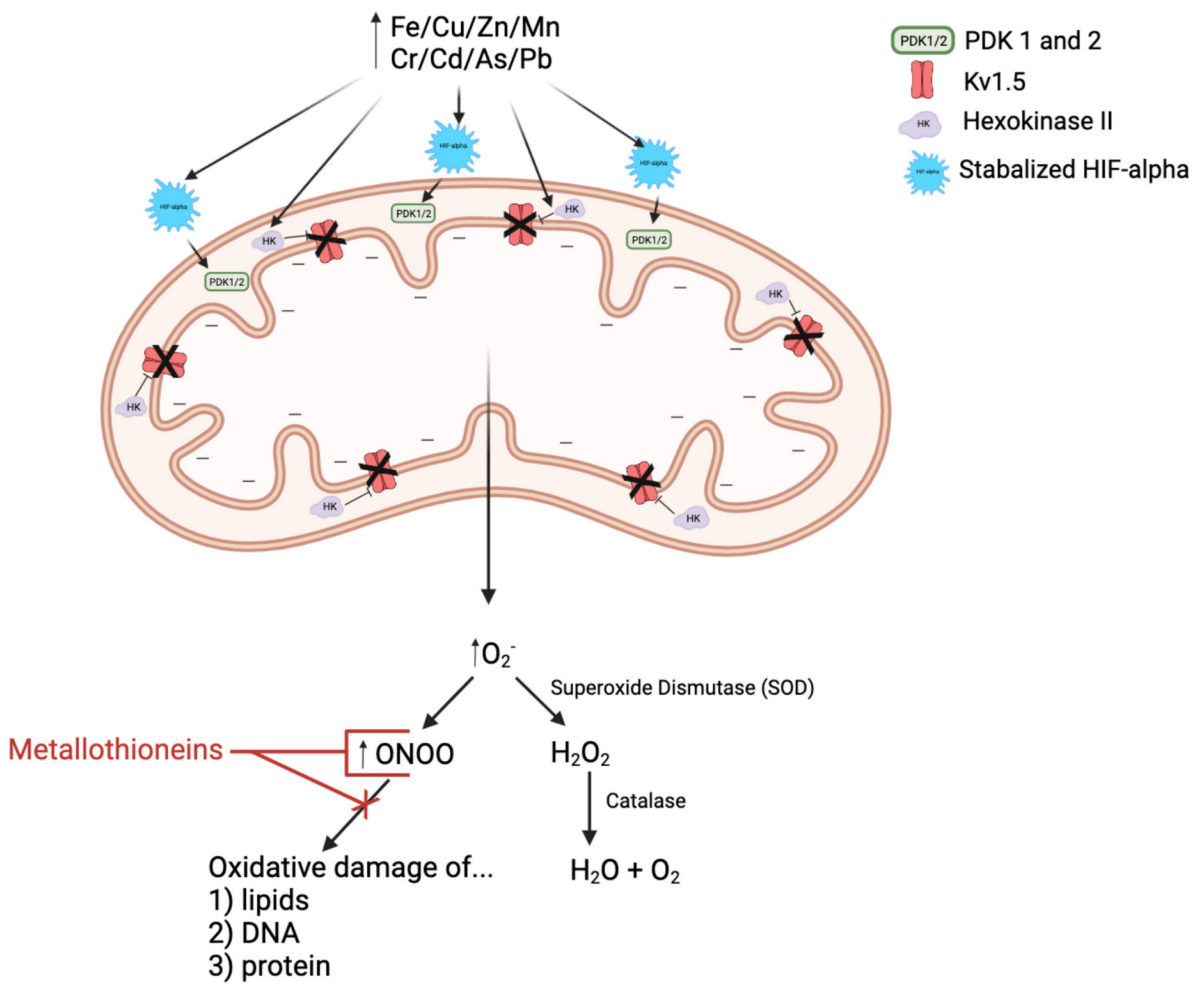
In response to the decreased oxygen availability associated with not only PAH but also heavy metal exposure, HIF-alpha is stabilized and thus activated. Stabilized HIF-alpha promotes translocation of hexokinase II into the mitochondria, thus inhibiting Kv1.5 activity and hyperpolarizing the mitochondrial membrane. Stabilized HIF-alpha also induces upregulation of pyruvate dehydrogenase kinases 1 and 2 (PDK 1/2), resulting in decreased pyruvate dehydrogenase (PDH) activity. PDH is an essential upstream enzyme in mitochondrial oxidative phosphorylation, and inhibition of this enzyme decreases oxygen reduction and consequently increases superoxide ion (O_2_^-^) production, i.e.: reactive oxygen species (ROS). O_2_^-^ is then either spontaneously interacts with nitric oxide (NO, reactive nitrogen species, RNS) to form more reactive RNS, peroxynitrite (ONOO) or is converted to another ROS called hydrogen peroxide (H_2_O_2_) via superoxide dismutase (SOD), which is then broken down into water and oxygen by the enzyme catalase. Metallothioneins (MTs) are cysteine-rich heavy metal and ROS/RNS scavengers. They have been shown to be beneficial in the treatment of PAH by binding to and reducing the activity of ROS/RNS, like ONOO, and thus limiting the oxidative damage of arterial lipids, DNA, and proteins.

As illustrated in **Figure 2**, many experiments showed that heavy metals can cause mitochondrial damage, impair ROS clearance system, increase ROS production, lead to lipid peroxidation, cell signal protein oxidation, and even DNA strand breaks. Increased intracellular ROS contents can destroy the intracellular membranes ion conversion channel, leak Ca^2+^ out, cause mitochondrial membrane dysfunctions, increase oxidant production, and damage the mitochondria. Impaired mitochondria display morphological and functional changes and produce more oxidants and less ATP. The integrity of the mitochondrial membrane is prerequisite for normal progression of oxidative phosphorylation and ATP generation. The integrity of the mitochondrial inner membrane is important because the electrochemical gradient and proton gradient on both sides of the membrane must be maintained 49, 50. In response to heavy metals, mitochondria increase Ca^2+^ intake, causes mitochondrial transient potential (mPTP) opening, increases mitochondrial membrane permeability, affects the imbalance of ion exchange causes, and alters membrane potential. The inner mitochondrial membrane contains enzymes involved in the electron transport chain and ATP production. The energy of most human cells is produced by the mitochondria. Heavy metals aggravate the oxidative damage of the mitochondria by reducing the activity of NADH and ATP enzymes and by affecting key enzymes, leading to suppressed ATP generation and a substantial increase in ROS 51, 52. The metals that damage mitochondrial structure and function include both non-essential metals such as Cd, Pb, Hg, and essential metals, including Ca, Zn, Fe, Cu 46, 53. Therefore, the mitochondrial effects by metals have been considered as the major pathogenic mechanisms for several diseases 52. However, whether metals-mediated mitochondrial damage occurs in pulmonary arteries and plays any role in the pathogenesis of PH remains largely unknown 7, 54-56. Therefore, the aim for this review is to assess the evidence surrounding essential and non-essential metals, mitochondrial dysfunction, and the subsequent ROS/RNS production in PH. Due to some similarities between SH and PH, the pathogenic effect of metals on SH will be briefly introduced before discussing metals in the pathogenesis of PH.

### 3.2 Effects of metals on systemic hypertension

The National Health and Nutrition Examination Survey (NHANES) defined SH as systolic BP ≥ 130 mmHg and diastolic BP ≥ 90 mmHg. A systematic review concluded that prolonged exposure to high levels of Pb might cause SH in adults 57, which was supported by a cross-sectional and prospective study showing the association between Pb levels and elevated blood pressure levels in a dose-response relationship 58, 59. This was consistent with the Strong Heart Study of Indigenous Americans showing a relationship between arsenic and non-linear increases in BP 60. The positive association of exposure to As with hypertension was supported by a recent meta-analysis based on a total of 27 studies comprising 117,769 participants, revealing that As exposure was mainly associated with increasing systolic blood pressure but not significantly related to diastolic blood pressure, and also confirming the nonlinear dose-response association 61.

Associations of metals with SH were also estimated for both single and multiple metal models in 11,037 adults from the 2017-2018 China National Human Biomonitoring. Higher rates of pre-hypertension (defined as systolic BP between 120 - 139 mmHg and diastolic BP between 80 - 89 mm Hg) and hypertension (systolic BP/diastolic BP ≥ 140/90 mmHg) were both associated with elevated blood Pb or As levels, but more remarkable with mixtures of metals in the blood 62.

The toxicity of inhaled Cd leads to inflammation and fibrosis, especially around blood vessels near the alveoli, and destruction of the lung immune barrier. Early epidemiological studies investigated the elements in rainwater and their relation to the frequency of hospitalization for SH, in Opole Voivodship, Poland during 2000-2002, revealing a mild correlation between Cd and cases of SH with some sex differences 63, 64. In 2017, the Strong Heart Study 65 showed an association between urinary Cd concentrations and systemic blood pressures in Indigenous Americans. Among 3714 middle-aged Indigenous American participants, urinary Cd levels at 0.01 - 78.48 μg/g creatinine were significantly associated with higher systolic BP in models adjusted for age, sex, geographic area, body mass index, smoking (ever vs never, and cumulative pack-years) and kidney function. Therefore, these studies indicated positive relationships between blood and hair Cd levels and SH. Hair is the optimal biological sample to identify the relationship between Cd exposure and SH for both genders 66 even though there was a contradictory report 4 and more studies were suggested 66.

Other metals' potential links to SH were relatively less investigated. Tungsten is an emerging contaminant in the environment primarily due to increased use of tungsten in industrial applications. Its potential impact on human health remains unknown 67, 68. However, the significant impact of tungsten on diabetes, metabolic syndrome, 69-71 and kidney diseases 72-74 have been reported by multiple studies. In fact, when Shiue and Hristova investigated the relationships of different sets of urinary environmental chemical concentrations and SH in a national, population-based study with the United States National Health and Nutrition Examination Surveys, 2009-2012, they indeed found a significant association between tungsten concentrations and SH 75. In addition, by examining urine metal levels of 823 patients in China, high urinary Hg level was also found to correlate with high diastolic BP 76.

In addition, it should be clear that the metals that are associated with SH are not only nonessential metals, in fact cross-sectional study in two rural areas of southwest China showed positive trends between SH and increasing quartiles of Fe in the Cd-polluted area and Mg, Ca and Cu in the unpolluted area. These findings suggest that exposure to Fe in the polluted area while Mg, Ca and Fe in the unpolluted area might increase the risk of SH or elevate blood pressure levels 77.

In summary, the harmful effects of metals on SH have been extensively reported via several mechanisms such as NO availability, upregulation of inflammatory pathways, and induction of ROS. Whether these metals may be contributors to the pathogenic effects of metals on the pulmonary vasculature, and whether exposure to nonessential metals disturb essential metal homeostasis that secondarily affects the pulmonary artery structures and induces PH, will be discussed in the following section.

## 4. Metals and PH

The pulmonary vasculature is one of the first capillary beds that encounters the environmental air pollution. The environmental pollution, tobacco smoking, and other particles may generate free radicals and damage vascular cells causing cellular dysfunction 78. Particulate matter (PM) contains heavy metals in addition to other toxic components 5. Occupational exposure is linked to increased health burdens caused by heavy metals. Although only few studies have focused on the direct correlation between high levels of pollution and PH, there have been some associations identified. Models of occupational exposure have been linked to pulmonary changes. Mice exposed to endotracheal silica developed PH and subsequent RV hypertrophy. Increased RV pressure, fibrosis, and granulomatous inflammation resembled the hallmark pathophysiology of PH-increased muscularization of the arteries leading to vascular occlusion. Inflammatory markers such as TNF-α, were upregulated in addition to markers of remodeling 79. Furthermore, emission-derived Zn-containing PM given intratracheally increased inflammatory markers in control, normotensive, and spontaneously hypertensive rats. Single exposure showed dose-dependent lung injury and inflammation, with the most dramatic increase in the spontaneously hypertensive rats. Despite no significant changes in neutrophilic inflammation with inhalational exposure of the emission-derived PM, high-dose exposure over 4 days showed a mild increase in lung permeability and particle deposition in alveolar macrophages 80.

Human studies also showed associations between heavy metals and PH. Workers in an open-pit Mn mine in Guangdong, China had worse lung functions than controls due to exposure to fine PM with a diameter less than 2.5 μm (PM_2.5_). These effects were attributed to proximity of workers to the dispersion of the PM_2.5_, particle size, and free silica content of the PM. Proposed damage to lungs with occupational exposure - intimal thickening, capillary damage, and permeability changes - were consistent with the changes seen in the mining workers 81. Occupational exposure in steel mills in Iran showed a higher incidence of PH and RV dilation in these workers 82. This is supported by previous studies on higher rates of RV dysfunction in workers exposed to PM_2.5_ as well as household exposures 83-85.

Many of the underlying diseases associated with PH are cardiopulmonary in nature. Certain CVDs which are risk factors for the development of PH, such as arrhythmias and heart failure, have been associated with fine PM exposure in air pollution 5. In our recent pilot study of 20 PAH patients and 10 healthy controls, multiple metals in whole-blood, plasma and urinary samples using an inductively coupled plasma mass spectrometry (ICP-MS) demonstrated in the plasma samples, silver and copper levels were significantly higher in PAH patients. There was significant positive correlation between cardiac output and cardiac index with plasma levels of silver. There was significant correlation between mixed venous saturation, 6-min walk distance, and last B-type natriuretic peptide with plasma levels of chromium 56. Furthermore, antimony (Sb) levels correlated with PAH severity 86. Therefore, while metal concentrations of PM can vary greatly, even relatively low concentrations of metals can produce dramatic effects in the cardiopulmonary system.

### 4.1 Non-essential metals and potential roles of its dyshomeostasis in PH and RV dysfunction

Study on the direct effects of non-essential metals on PH is relatively lacking. A summary of the contribution of heavy metals to the pathophysiology of PH is shown in **Table 3**.

#### Lead

Pb has toxic effects on many visceral organs and disrupts physiological functions. Pb levels and its distribution differs in certain organs, and accumulated Pb levels are particularly higher in the cerebral cortex and lungs 87. Different levels of Pb accumulation correlate with the degree of disruption. From PH perspective, Pb has been shown to alter the activity of vascular smooth muscle via the depressed production and sequestration of NO within the vasculature, specifically through increased production of nitrotyramine 88, 89. In rodent models, the involvement of Pb in the production of ROS/RNS and oxidative damage, cytotoxicity, cellular dysregulation, and reduced NO effects was attributed to hypertension and tissue damage 88.

It is reported that 7-day exposure to Pb increased vascular dysfunction by limiting contractile and relaxing functions of rat pulmonary arteries. In Pb-treated rats, ROS were found to be increased: for example, superoxide was higher when compared to controls. Although there was an increase in NO, the vascular smooth muscle did not respond adequately to the increased NO, indicating a dysfunction in the vasodilatory response. There is a need to evaluate the mechanism of NO resistance in the pulmonary vessels 90. As discussed by Bhatnagar 5 and Briffa, et al. 6, chronic Pb exposure is associated with decreased NO availability, thus disrupting the endothelial cell signaling mechanisms responsible for vasodilation. This lack of vasodilation increased systemic and pulmonary pressures consistent with the effects seen from increased heavy metal exposure via air pollution. In addition, Pb also disrupts Fe regulation and accumulation via the reduction of heme synthesis, and indirectly leads to more Fenton reactions and oxidative stress (See Section 4.2 for further discussion of Fe's role in PH).

In summary, Pb exposure causes systemic and pulmonary damage via multiple mechanisms, including increased oxidative damage, disruption of heme synthesis, and dysregulation of vasodilatory mechanisms.

#### Cadmium

Cd has wide-ranging effects on multiple key enzymes in oxidative phosphorylation and components of endothelial barriers, leading to increased vascular permeability. In *ex vivo* bovine pulmonary arteries, Cd and Hg toxicity led to dysfunction via ROS accumulation 91. Oxidative damage and increased ROS have been linked to hypertension, however it is unclear whether oxidative damage causes increased blood pressure, or is a consequence of the pathophysiology of hypertension as ROS mediate vasoconstriction and vascular remodeling via several mechanisms.

Analysis of 8700 subjects from the Third National Health and Nutrition Examination Survey (NHANES III) pointed out that adult smokers with low dietary Zn intakes have a higher Cd burden and increased risk of developing chronic obstructive pulmonary disease (COPD) 92. Although COPD does not directly link to PH, mild-to-moderate PH is a common complication of COPD. Therefore, Cd exposure via smoking increases the risk of COPD and may also indirectly increase the risk of PH.

A case-control study supported the above notice, by demonstrating associations of circulatory Cd and COPD among 400 COPD patients and 400 control subjects. Circulatory Cd was inversely associated with pulmonary functions in COPD patients. Meanwhile, E-cadherin, one of epithelial biomarkers, was reduced in lung tissues of COPD patients with higher circulatory Cd, which was accompanied with reduced levels of pulmonary N-cadherin, Vimentin and α-SMA, three mesenchymal biomarkers 93. A recent study used the US-based Registry to Evaluate Early and Long-term PAH Disease Management (REVEAL) to assess the prevalence, demographics, and outcomes in ever- versus never-smokers with PAH. Smoking prevalence in male PAH patients is disproportionate. The prevalence of cigarette smoking was significantly higher in males than females. Ever-smoking status was associated with increased age at PAH diagnosis and, in newly diagnosed male PAH patients, earlier time to hospitalization and shorter survival after PAH diagnosis 94. However, whether these unclear associations between smoking and PAH are related to Cd remains largely unclear. More direct effects of Cd on the components of pulmonary artery such as PAECs and PASMCs and RV myocytes urgently need to be investigated.

Exposure to Cd leads to vascular remodeling via many mechanisms, including increased smooth muscle cells, NOS dysregulation, and ROS production 95, 96. As stated, lower levels of NOS (and therefore NO) are associated with the pathogenesis of PAH via vasoconstriction, inflammation, and thrombosis formation. Cd-treated rodents showed increased fibrotic responses in rat aortic tissues along with increases in O^2-^, urinary nitrate/nitrite, MDA, carbonyl level and decreased GSH production in rat blood and thoracic aorta tissues, leading to the arterial remodeling and stiffness, development of high arterial blood pressure, and cardiac dysfunction 96. Exposure to ambient PM_2.5_ and PM_10_ in juvenile, adult and senescent rats for 12 months with the average concentrations of 78.7 PM_2.5_ or 128.2 μg/m^3^ PM_10_ could destroy intrapulmonary and small artery endothelial functions, causing vasodilatory disorders. In these rats, PM_2.5_ and PM_10_ exposure caused PM to accumulate in the lungs, along with Cd accumulation in the liver and kidneys. Therefore, severe pulmonary and vascular disorders caused by ambient PM_2.5_ and PM_10_ might be related to Cd in PM 97.

One question is whether Ca is required for Cd-mediated pathogenesis of PH since intracellular Ca ion concentration (Ca2+) was associated with PH-related vascular remodeling and vasoconstriction 98. Reportedly, Cd mobilized cellular Ca2+ in human skin fibroblasts. The divalent metals produced a large spike in cytosolic free Ca2+ and strikingly increased net Ca2+ efflux similarly to bradykinin. Zn2+ competitively and reversibly inhibited net Ca2+ efflux produced by Cd, but not that produced by bradykinin 99. Similarly, Cd evoked Ca2+ mobilization in the fibroblasts of umbilical artery muscle and endothelial cells 100, 101. They found that Cd at very low-dose levels (≤ 100 nM) can increase the incorporation of thymidine into the acid-insoluble fraction of growing bovine and rabbit aortic smooth muscle cells but not of growing bovine aortic endothelial cells, suggesting that low levels of Cd may promote the proliferation of vascular smooth muscle cells through intracellular Ca2+dependent signaling pathway 101. Pre-incubating the cells with Zn2+ strongly inhibited the Cd stimulation of Ca2+ efflux without affecting the stimulation of efflux by ATP, suggesting that certain trace metals trigger the release of stored Ca2+ by stimulating a cell surface “receptor” 100.

In addition, platelet-derived growth factor (PDGF) also increases intracellular free Ca2+ in human dermal fibroblasts. However, PDGF stimulating effect was markedly inhibited by genistein that selectively inhibits tyrosine kinases. Genistein did not prevent Cd-stimulated Ca2+ mobilization into cytosol. These suggest that several stimuli such as Cd and PDGF can stimulate Ca2+ efflux or rapid increase at intracellular level through different signaling pathways. However, whether stimulation of intracellular Ca2+ result in the same pathogenic effects also depended on the status of intracellular Ca2+ clearance by plasma membrane Ca-ATPase (PMCA) 102. Emerging evidence indicates the important pathogenic effect of PDGF-BB on PH 103-105. Since HIF-1 upregulation is the key response to PH, its down-stream genes' upregulation plays major pathogenic roles in PH-associated pathogenesis and RV dysfunction. As one of HIF-1α -downstream genes 106, 107, PDGF-BB was up-regulated in response to hypoxia. In PASMCs of PAH models, expression of PDGF-BB significantly increased along with increased intracellular Ca2+ and down-regulation of PMCA4 expression 108. Whether Cd has certain inhibiting effects on PMCA4 in the PAECs or PASMCs of PAH models remains unclear, but Cd induced erythrocyte toxicity was also found to be associated partially with its inhibition of PMCA that mediates Ca2+ extrusion 109. Cd's sperm toxicity was also associated with its inhibition of PMCA 110. These suggest similar mechanisms might exist in PH by which exposure to Cd or hypoxia upregulates PDGF-BB and causes pulmonary vascular cell and RV myocytes remodeling.

Although exposure to chronic Cd may cause CVDs, including hypertension from the vascular endothelial cell damage, the matter of whether exposure to Cd directly induces or only exacerbates PH and RV remodeling and dysfunction remains unclear and warrants further investigation.

#### Antimony

Antimony is a chemical element and its air pollution and/or environmental contamination is derived from its wide application and associated products 111. Since the 1940's, Sb toxic effects on cardiovascular system were noticed. The first link between Sb and hypertension was a study on the urinary excretion of metals including Sb in five patients with hypertension before and during treatment with hydralazine 112. Consequently, two studies from NHANES 2009-2010 113 and 2009-2012 [75]showed urine Sb with other metals (urine cobalt, Pb, and tungsten) was significantly associated with hypertension. Similar findings were subsequently reported 114-116.

In our recent pilot study, we evaluated Sb levels in the blood, plasma and urine of 20 PAH patients and 10 controls, which showed significantly higher blood and plasma levels of Sb in PAH patients when compared to controls. Sb blood and plasma levels were also significantly higher in both IPAH patients and non-IPAH when compared with controls. But these differences were not significantly observed in the urine levels of Sb. In addition, there was a significant correlation between plasma Sb levels and all the PAH prognostic hemodynamic parameters, including mean right atrial pressure (mRAP), CO, CI, PVR, and mixed venous oxygenation (SvO_2_) 86.

#### Vanadium

Vanadium (V) is in the first transition series of the periodic table, specifically in the 5 group states. V is omnipresent in trace amounts in the environment and atmosphere. V is also present in the human body, where it might serve as a regulator for phosphate-dependent proteins. The unique chemical and physical features of V compounds make it a widely required material in many industries and medical fields. The wide use of V also leads to its release to, and therefore presence in, the environment. Occupational exposure to V was found to induce, e.g., acute respiratory symptoms in boiler makers 117, 118.

Earlier studies have reported that exposure to V oxides attached to fine PM_2.5_ was associated with increased risk of respiratory symptoms in children 119 and older people in US counties 120. Two recent studies, based on Chinese populations, showed the association of V exposure with SH and BP levels 121, 122. However, it remains unclear whether V can directly induce PH in human. The direct roles of V in the pathogenesis of PH have been examined with animals exposed to V. Rats treated with dietary V for 2 months developed PH, as indicated by significantly higher PVR mean and systolic RV pressure without significant effects on CO when compared to rats without V treatment. RV hypertrophy was also noted in V-treated animals. The results indicate that V administration induces PH 123. This was further confirmed and defined that V induced acute pulmonary vasoconstriction was partially through the inhibition of endothelial NO production via PKC-dependent phosphorylation of Thr495 of eNOS 124.

#### Other metals

As mentioned above, the potential link of tungsten to SH has been implicated 113. However, whether tungsten is linked to PH has not been directly addressed even though its general pulmonary toxicity has been demonstrated 68, 125, 126. Liu et al. 127 developed tungsten-based polyoxometalate nanodots (WNDs) with potent elimination of multiple ROS due to its high proportion of reduced W5+. Treatment of PAH rats induced by monocrotaline with intravenous injection of WNDs significantly prevented the abnormal proliferation of PASMCs, greatly improved the remodeling of pulmonary arteries, and ultimately improved RV function. Although this study did not address whether tungsten contributes to PH, it shed lights on future studies on the impact of tungsten on PH/PAH.

In summary, we did not have direct evidence to indicate the causative role of these non-essential metals mentioned above in the pathogenesis of PH. However, whether exposure to these metals would worsen the outcomes of PH should be urgently investigated.

### 4.2 Essential metals and the potential role of its dyshomeostasis in PH and RV dysfunction

Essential metals are required for the biological functions, but their dyshomeostasis (i.e.: either too low [deficiency] or too much [excessive]) causes vascular injuries or remodeling, contributing to the development of PAH, RV remodeling, and cardiac dysfunction. These metals play key roles in NO metabolism dysregulation and production of ROS/RNS and free radicals. Metabolic disturbances, mediated by Cu ions, can induce DNA strand disruptions and oxidation of the DNA bases. Furthermore, Cu and Fe undergo Fenton reactions to produce free radicals 6, 44, 45. These metals often correlate with one another and when in combination with essential and nonessential heavy metals, essential heavy metals can worsen the toxic effects and reinforce persistent cycles of inflammatory responses 45, 128. A summary of the contribution of these heavy metals to the pathophysiology of PAH can be found in **Table 3**.

#### Copper

Copper is a major component of non-exhaust traffic-related pollution. It is taken up by membrane protein Cu transporter (CTR1). Once absorbed, Cu serves as a critical element in the redox reactions necessary for hemoglobin synthesis, protective functions for DNA, and nervous and immune system functions 6, 44, 128-130. Through these reactions, cuprous ions form from the reduction of cupric ions. This reduced form of Cu can then produce superoxide and hydroxyl radicals via the Fenton reaction and these highly reactive species are harmful to proteins, lipids, and DNAs 6, 44.

In Toronto, long-term exposure to Cu with Fe was associated with increased ROS in lung epithelial fluid and worse mortality from respiratory diseases. COPD mortality was associated with higher levels of Cu exposure 131. So, despite the relatively small proportion of Cu and Fe in PM_2.5_, the long-term health effects may be great though more research is needed to elucidate these mechanisms 131, 132. Additionally, Cu can form complexes with homocysteine that have been suggested to induce vascular injury and endothelial cell dysfunction 133. Furthermore, Cu and ceruloplasmin (a Cu storage protein) have been correlated with the inflammatory conditions such as systemic sclerosis. In sclerotic patients with and without PAH, ceruloplasmin was elevated in comparison with normal controls, indicating Cu's role in inflammatory processes 52, 128. In addition, serum levels of Cd, Co, and Fe were found to be higher in COPD cases with PH compared to COPD patients without PH 128.

Mechanistically, Cu is a producer of ROS, but it also plays a role in anti-oxidative stress as Copper-Zinc superoxide dismutase (CuZn-SOD) 6, 128, 130. Cu is delivered to CuZnSOD via a chaperone by the CTR1 transporter. Cu is also transferred to a Cu-transporting P-type ATPase (ATP7A) via COX 17 (mitochondria) and ATOX 1 (Golgi). By transporting Cu to the Golgi apparatus, it can be used to synthesize proteins such as pro-Cu-dependent lysyl oxidase (LOX). Pro-LOX is cleaved by metalloproteinases into LOX which is involved in collagen and elastin crosslinking in the vascular wall. Both LOX and CuZn-SOD are implicated in vascular changes seen in PAH. Upregulated activity of LOX may lead to vascular stiffening. Dysregulation of CuZn-SOD leads to increased ROS and oxidative damage. High Cu levels in normal physiology leads to increased Cu efflux via ATP7A. However, under chronic hypoxic conditions, upregulated CTR1 transporter, ATP7A, and pro-LOX are noticed in animal models of hypoxic PH and in human lung tissues. Chronic low oxygen levels activate HIF-1α which leads to the upregulation of CTR1 and proliferation/migration of PASMCS. Increased Cu uptake may then stabilize HIF-1α and exert pro-inflammatory effects 130.

Cu interacts with other heavy and essential metals. Cu deficiency can contribute to Zn toxicity and both Cu and Zn can interfere with Fe metabolism. In fact, Zn supplementation can alter the intestinal absorption of Cu and cause anemia 6, 44, 128, 133. Therefore, Cu is not only independently associated with risk factors for PAH, but also contributes to heavy metal toxicity along with other heavy metals, as discussed above.

#### Iron

Iron is an essential metal and involved in metabolic processes such as electron transport, nucleotide synthesis, and oxygen transport. Although essential for metabolism, Fe can be toxic if concentrations exceed a certain threshold. Therefore, Fe levels need to be held in tight regulation. In the human body, Fe is concentrated in hemoglobin, bound to ferritin or transferrin, embedded within intracellular proteins, and in a labile iron pool. This labile Fe pool, also referred to as non-transferrin bound Fe, is a reactive pool of relatively free-floating Fe. Iron in this non-bound state can form ROS, in this case a free radical as Fe is transformed from its ferrous (Fe^2+^) to ferric (Fe^3+^) state 134.

Although Fe metabolism is regulated by many factors, of particular interest to PH is its regulation by hepcidin. Hepcidin is a 20-25 residue peptide that binds to ferroportin, preventing Fe absorption from enterocytes and promoting Fe utilization within the body. Fe deficiency induces the expression of IL-6, and IL-6 likewise stimulates the expression of hepcidin via the STAT3 pathway. One crucial consequence of PAH is hypoxia 134. HIF-2α expression increases in response to hypoxic conditions and HIF-1 regulates mammalian oxygen homeostasis by transcriptionally activating the expression of numerous target genes. Fe regulates HIF expression via iron response proteins (IRPs) binding to iron response elements (IREs) within HIF mRNA 135. HIF-2α expression induces PDGF, erythroferrone, matriptase-2, and growth differentiation factor 15; all of which suppress hepcidin expression 136, 137. Hepcidin-KO mice showed increase accumulation of Fe in the alveoli of the lungs 137. Conversely, elevated hepcidin levels result in decreased duodenal Fe absorption and dampened stress-mediated erythropoiesis 138. This leads to systemic Fe deficiency, but intracellular Fe overload, and causes ROS production via mitochondrial dysfunction. Additionally, Fe overload in cells disturbs lipid peroxidation which can affect protein handling and pulmonary vascular remodeling 139.

Therefore, abnormal expression of any enzymes or proteins required for Fe metabolism will affect Fe homeostasis. NFU1 is a mitochondrial protein that is involved in the biosynthesis of Fe-sulfur clusters, and its genetic modification is associated with disorders of mitochondrial energy metabolism. Most of patients with NFU1 mutation G208C develop PAH 140, 141, consistent with rats carrying mutant human G208C developed typical PAH. Importantly, the penetrance of the PAH phenotype was found to be more prevalent in females than in males, replicating the established sex difference among patients with PAH 142.

In terms of how Fe affects the cardiovascular system and lungs, Fe can reduce NO production by reducing transcription of inducible NOS (iNOS) 134 and the labile Fe pool can contribute to ROS/RNS production via Fenton reactions 20. The association between Fe overload and PH may be implicated by the fact that the patients with thalassemia syndromes have higher risk to develop PH 143, 144 and PH was almost absent in patients with thalassemia major if they strictly adhered to standard transfusion with iron chelation therapy 143, 145. Furthermore, this link was evidenced by a study with animal models, where administration of Fe chelator, deferoxamine, to rats prevented chronic hypoxia-induced PH and pulmonary vascular remodeling 146. Besides Fe overload, Fe deficiency was also associated with PH. Intracellular Fe deficiency was associated with a thicker PASMC layer, increased RV systolic pressure, and reduced RV ejection fraction in a PH mouse model 147. The percentage of Fe deficiency is as high as 63% in IPAH patients 139. In PAH, ROS/RNS act as secondary messengers for the growth of PASMCs during cellular signaling, thus triggering RV hypertrophy and pulmonary vascular remodeling. Fe-dependent protein carbonylation is promoted by the presence of ROS/RNS, thus initiating this intracellular signaling cascade 20.

It is worth noticing that despite the connections between Fe homeostasis and PH, interventions targeting Fe pathways in PH patients have shown varying results. Application of Fe chelation as a main therapeutic approach for the patients with PH or PAH are not clinically applied. However, recent summary revealed that oral Fe supplementation with traditional formulation showed limited utility due to poor absorption. Newer oral formulations such as ferrous maltol and pyrophosphate sucrosomial Fe, with intrinsically improved bioavailability, have demonstrated possible clinical benefits, including improved exercise capacity measured by 6-min walk distance 148. Therefore, more molecular mechanisms for the pathogenesis of PH or PAH are urgently needed, one of which may include the role of ferroptosis that has been discussed and may provide new insights 149-151.

#### Zinc

Zn is a necessary component of many enzymes and play key roles in the enzymatic activity of alcohol dehydrogenase, DNA polymerases, and RNA transcriptase which highlights Zn's role in synthesis and maintenance of nucleic acids 6, 128. Zn has been noticed for its contribution to lung epithelial toxicity *in vitro* 44. At normal concentrations, Zn serves an anti-inflammatory and antioxidant role, due to its ability to protect against oxidative stress caused by other metals 6, 44. At elevated concentrations, however, Zn itself can contribute to the formation of ROS/RNS as well as interfere with Cu and Fe absorption and metabolism. High extracellular Zn activates MAPKs, leading to inflammation and trigger apoptosis 6. In fact, acute and large exposure to Zn has been implicated to induce PAH since two men, fatally injured by accidental inhalation of Zn chloride from a smoke bomb, have quickly developed lethally PH 152. High level of blood Zn-protoporphyrin along with Fe deficiency was also found in patients with IPAH 153. Therefore, maintaining intracellular Zn homeostasis is very important to maintain normal cell and organ function.

**Figure 3:**
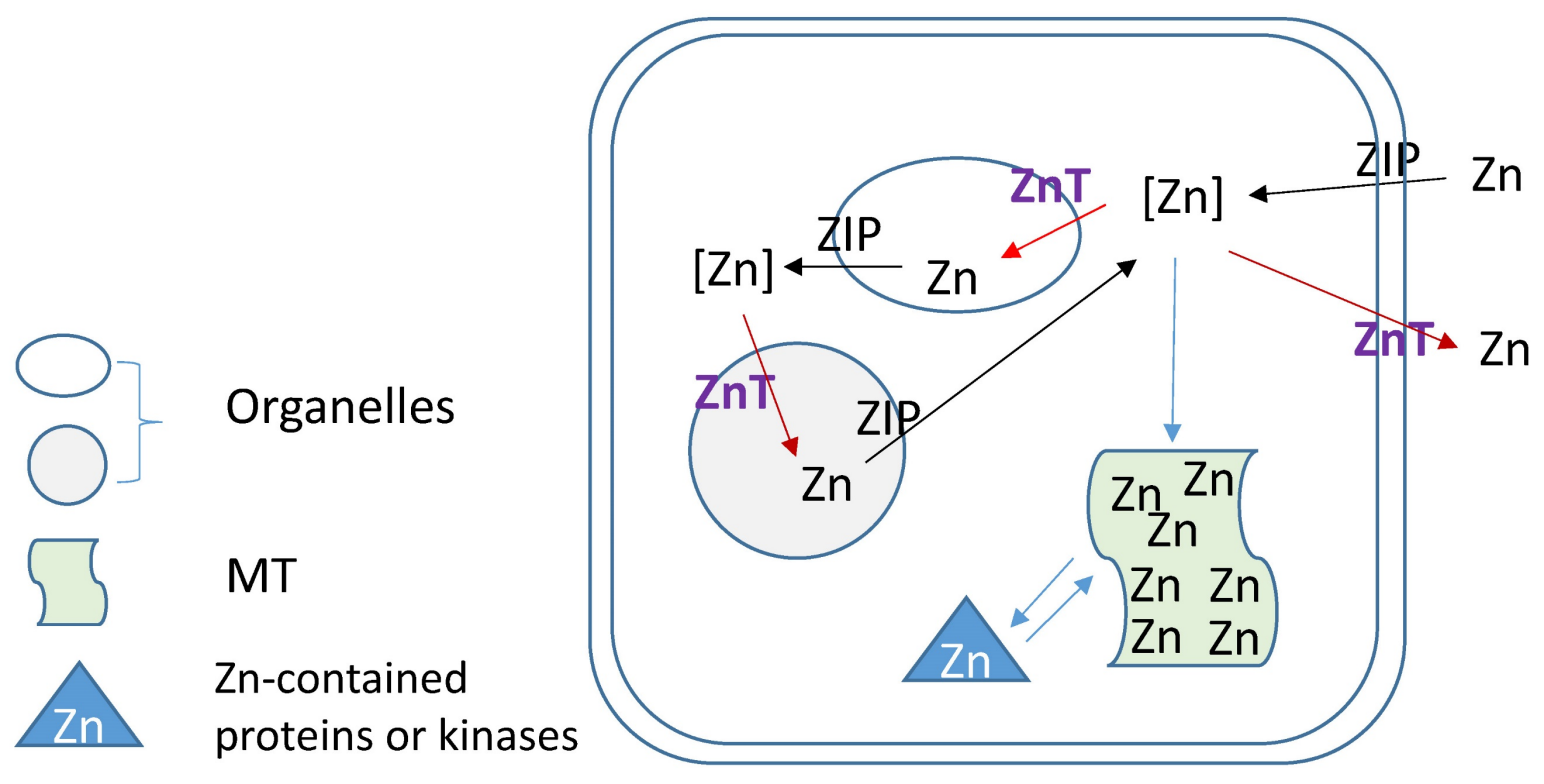
Zinc regulation by its transporters (ZIPs and ZnTs) and metallothionein (MT).

Zn homeostasis is known to be regulated by different proteins involved in uptake, excretion and intracellular storage of Zn. These proteins belong to the ZIP (Z̲rt- and I̲rt-like proteins (solute-linked carrier 39, SLC39A) and ZnT (solute-linked carrier 30, SLC30A) families as well as MTs 154, 155. Members of the ZnT family (ZnT1-10) reduce intracellular Zn concentrations by transporting free Zn out of the cytosol or into intracellular vesicles while members of the ZIP family (Zip1-14) are responsible for Zn uptake from the extracellular space or release from intracellular compartments, as illustration of **Figure 3**. MTs as Zn storage and donor play important roles to bind free Zn and release its bound Zn to the Zn-contained proteins or kinases for their stable or functional conformation. As one of ZIP family members, ZIP12 has been appreciated for its expression and role in the nervous system. Normally, this transporter is undetectable in normoxic pulmonary tissues and cells; however, in hypoxic environments a marked increase in ZIP12 mRNA expression is present in multiple cell types related to pulmonary vascular remodeling, including vascular smooth muscle, endothelial, and interstitial cells 156. This specific response of ZIP12 to hypoxia is because of the enrichment of hypoxia response element for both HIF-1a and HIF-2a on ZIP12 gene promoter. In addition, the response of ZIP12 to hypoxia was significant in other species, such as cattle and human, showing increased expression of ZIP12 protein when under hypoxic conditions; in the lung tissues from chronic Fe-deficient rats; rats exposed to monocrotaline (MCT), as well as in patients with IPAH 156. Measuring intracellular Zn level showed the parallel increase with ZIP12 protein expression, suggesting the increased ZIP12-mediated intracellular Zn uptake 156. Inhibition of ZIP12 expression significantly or partially prevented hypoxia-induced PAH, RV hypertrophy and vascular remodeling than wild type rats, suggesting the essential role of ZIP12 expression in the pathogenesis of PH. This important finding was further supported by the subsequent studies with different conditions 157, 158.

## 5. Conclusions

Currently, there are few definitive mechanisms identified in PH pathophysiology and even fewer efficacious treatments available for PH. Many factors (clinical, hemodynamic, imaging, etc.) are used to determine a treatment plan for each patient. However, metal levels and exposures are not evaluated comprehensively 159-161. Little information is available on the exact mechanisms surrounding metals and their potential involvement in PH. Metals have strong correlations to the vascular and cardiac remodeling, metabolic dysfunction, and mitochondrial disorder, all of which are characteristics of PH, especially when associated with other cardiopulmonary pathologies, such as COPD. The potential roles of non-essential and essential metals in PH are further supported by MTs' cardioprotective functions and the association between iron deficiency anemia and PH. These key points are summarized in **Table 1** and major findings are summarized in **Table 4**. Promising evidence around MTs and other free radical-scavenging treatments suggest that these are possible avenues for future PH treatment.

Based on the above indirect clinical evidence and preclinical studies with animal models and *in vitro* cultured cells, we hypothesize that exposure to metals including cadmium and antimony may either directly cause PH, or enhance the risk of developing PH, indirectly accelerating the pathogenesis of PH. Future research should focus on comprehensive clinical metallomics study in PH patients, mechanistic evaluations to elucidate roles of various metals in PH animal models, and novel therapy clinical trials targeting metals. These important discoveries will significantly advance our understandings of this rare yet fatal disease.

Through the investigation of metal impact on SH, it has been clear that exposure to metal cluster (multiple metals) are more significantly associated with CVDs 162 and SH 62. Although there was no direct evidence for the association of exposure to multiple metals with PH, there was a strong association of antimony with PAH and its associated RV pathogenesis 86. Therefore, investigation for the potential role of metal exposure in the pathogenesis of PH, exposure to mixed metals should be considered for the epidemiological and preclinical studies 56.

## Figures and Tables

**Table 1:** Comparisons between SH and PH

	SH	PH
**Definition**	• High blood pressure in the arteries that supply blood to the body's tissues and organs. • Affects the systemic circulation and the left heart (blood vessels throughout the body).	• High pressure in the pulmonary arteries of the lungs affecting the right heart. • Primarily affects the pulmonary circulation (blood vessels in the lungs).
**Causes/Etiology**	• Lifestyle factors (e.g., unhealthy diet, lack of exercise), • Genetics • Age • Chronic diseases (e.g., obesity, diabetes).	• Certain lung diseases (e.g., chronic obstructive pulmonary disease, idiopathic pulmonary fibrosis), • Heart diseases, • Blood clotting disorders, • Connective tissue disorders. • Induced by endothelial damage, oxidative stress, chronic inflammation, immune dysregulation • changes in genetic expression, molecular pathway alteration, mitochondrial dysregulation and apoptosis resistance.
**Symptoms**	• Often asymptomatic • Symptoms such as headaches, dizziness, chest pain, shortness of breath, visual changes.	• Shortness of breath, fatigue, chest pain, dizziness, fainting, swelling in the legs and ankles, bluish lips or skin (in severe cases).
**Diagnosis**	• Blood pressure measurement, • Medical history review, • Physical examination.	• Echocardiogram, • Pulmonary function tests, • Right heart catheterization, • Imaging studies (CT scan, MRI) • Blood tests.
**Complications**	• Heart disease, • Stroke, • Kidney damage, • Vision loss, • Metabolic syndrome.	• Right heart failure, • Heart arrhythmias, • Blood clots in the lungs, • Heart valve problems.
**Treatment**	• Lifestyle modifications (healthy diet, regular exercise, stress management), • Medications (e.g., diuretics, beta-blockers, Angiotensin-Converting Enzyme (ACE) inhibitors).	• Treating the underlying causes, • Vasodilators that target different pathways including prostacyclin, endothelin, nitric oxide pathways, • Calcium channel blockers, anticoagulants, oxygen therapy, • Lung transplantation (in severe cases).

**Table 2:** General categories of metals based on its biological needs and metal atomic weight

	Essential metals 44	Non-essential metals^44^
	Sodium (Na) Potassium (K) Calcium (Na) Magnesium (Mg)	
**Heavy metals 6**	Manganese (Mn) Iron (Fe) Cobalt (Co) Copper (Cu) Zinc (Zn) Molybdenum (Mo)	Antimony (Sb) Chromium (Cr) Nickel (Ni) Arsenic (As) Silver (Ag) Cadmium (Cd) Mercury (Hg) Lead (Pb) Titanium (Ti) Vanadium (V) Gold (Au) Tin (Sn) Platinum (Pt)

1) A list of heavy metals according to their density of is greater than 5 g/cm^3^ and is more common in daily life 6. 2) Red font indicates transition metals 6.

**Table 3:** Summary of the heavy metals and their potential contributions to PAH pathogenesis.

	Levels in PAH	Contribution to PAH Pathology
**Lead**	Elevated	Disrupt heme synthesis, cause renal glomerular damage, decrease B2R expression, increase ROS production, decrease NO synthesis
**Cadmium**	Elevated	LDH leakage, increase vascular membrane permeability, cause glutathione depletion, inhibit G6PDH and GAPDH
**Antimony**	Elevated	Correlation with PAH hemodynamic parameters and RV dysfunction
**Vanadium**	Elevated	Partially due to inhibition of endothelial NO production via PKC-dependent phosphorylation of Thr495 of eNOS
**Copper**	Elevated	ROS production, endothelial cell dysfunction, anemia
**Iron**	Decreased	Anemia, mitochondrial dysfunction, increase RVSP, thicken PASMC layer
**Zinc**	Elevated	Loss of antioxidant properties, inhibit PDE, activate MAPK

**B2R**=beta 2 adrenergic receptor,** ROS**=reactive oxygen species, **NO**=nitric oxide, **LDH**=lactate dehydrogenase, **G6PDH**=glucose 6 phosphate dehydrogenase, **GAPDH**= glyceraldehyde 3 phosphate dehydrogenase, **RVSP**=right ventricular systolic pressure, **PASMC**=pulmonary artery smooth muscle cell, **MAPK**= mitogen-activated protein kinase.

**Table 4:** Summary of Major Findings and Conclusions

Major Findings	Heavy metals induce vasoconstriction, endothelial damage, and oxidative stress. Heavy metals interfere with NO metabolism and bioavailability, metabolic pathways, cellular biochemistry. Lead induces renin-angiotensin-aldosterone system (RAAS) and sympathetic tone contributing to indirect stress in the pulmonary circulation. Reduced form of copper is associated with cytotoxic properties through ROS formation. Iron dysregulation is implicated in altered gene expression, NO metabolism, and cellular signaling pathways. Iron deficiency is implicated in patients with PAH, suggestive of a role in vascular remodeling. Zinc homeostasis balances intrinsic enzyme activities and metabolic pathways. Higher zinc levels induce the formation of ROS and induction of cellular apoptosis. Novel evidence of metallothioneins confer protective properties against heavy metal toxicity and ROS scavenging
**Conclusions**	Evidence suggests a potential role for heavy metals in the pathophysiologic process of PH through metabolic dysregulation and mitochondrial dysfunction leading to cardiopulmonary vascular remodeling, which may offer further avenues of study in the pathophysiology of and treatment options for PH.

## Abbreviations

6MWD: 6-minute walk distance
ATP: Adenosine triphosphate
ALK1: Activin A receptor-like kinase I
BOLA3: BolA family member 3
BMPR2: Bone morphogenetic protein type 2 receptor
CCB: Calcium channel blockers
CVD: Cardiovascular disease
CAT: Catalase
CAV1: Caveolin-1
CBF/NF-Y: CCAAT-binding factor/nuclear factor-Y
COPD: Chronic obstructive pulmonary disease
CTEPH: Chronic Thromboembolic pulmonary hypertension
CAD: Coronary artery disease
cAMP: Cyclic adenosine monophosphate
cGMP: Cyclic guanosine monophosphate
DBP: Diastolic blood pressure
eGFR: Estimated glomerular filtration rate
EIF2AK4: Eukaryotic translation initiation factor 2 alpha kinase 4
FGF-2: Fibroblast growth factor-2
FPHN: French Pulmonary Hypertension Network
GATA4: GATA binding protein 4
G6PDH: Glucose-6-phosphate dehydrogenase
GAPDH: Glyceraldehyde-3-phosphate dehydrogenase
HIF: Hypoxia-inducible factor
iPAH: Idiopathic pulmonary arterial hypertension
IL: Interleukins
IREs: Iron response elements
IRPs: Iron response proteins
MnSOD: Manganese superoxide dismutase
mPAP: Mean pulmonary arterial pressure
MMP: Matrix metalloproteinases
MT: Metallothionein
miRNA: MicroRNA
MAPK: Mitogen-activated protein kinases
MAO-A: Monoamine oxidase-a
SMAD: Mothers against decapentaplegic homolog
NOX: NAD(P)H oxidase
NO: Nitric oxide
NOS: Nitric oxide synthase
PM: Particulate matter
ONOO: Peroxynitrite
PDE5: Phosphodiesterase 5
Kv: Potassium channels
KCNK3: Potassium Two Pore Domain Channel Subfamily K Member 3
PKC: Protein kinase C
PKG: Protein kinase G
PAH: Pulmonary arterial hypertension
PAP: Pulmonary artery pressure
PAWP: Pulmonary arterial wedge pressure
PASMCs: Pulmonary artery smooth muscle cells
PH: Pulmonary hypertension
PHORA: Pulmonary Hypertension Outcomes Risk Assessment
PH-ILD: Pulmonary hypertension with interstitial lung disease
PVR: Pulmonary vascular resistance
PDH: Pyruvate dehydrogenase
PDK1/2: Pyruvate dehydrogenase kinases 1 and 2
RNS: Reactive nitrogen species
ROS: Reactive oxidative species
REVEAL: Registry to Evaluate Early and Long-term PAH Disease Management
RAA: Renin-angiotensin-aldosterone
RV: Right Ventricle
5-HT: Serotonin
SOD: Superoxide dismutase
SPAHR: Swedish Pulmonary Arterial Hypertension Registry
SBP: Systolic blood pressure
TRP: Transient receptor potential
REVEAL: US Registry to Evaluate Early and Long-term PAH Disease Management
VEGF: Vascular endothelial growth factor
WU: Woods Units
